# Spontaneous Onset of Postoperative Chilaiditi Syndrome: A Case Report and Its Potential Other Association?

**DOI:** 10.14740/jmc5249

**Published:** 2026-03-04

**Authors:** Ridwan Hashi, Khaled Elmagraby, Hannah Faherty, Daniel Huntley, Mohamed H. Ahmed

**Affiliations:** a Department of Surgery, Norforlk and Norwich University Hospital, Norwich NR4 7UY, UK; b Department of Acute Medicine, Milton Keynes University Hospital NHS Foundation Trust, Eaglestone, Milton Keynes, Buckinghamshire, UK; c Department of General Surgery, Milton Keynes University Hospital NHS Foundation Trust, Eaglestone, Milton Keynes, Buckinghamshire, UK; d Department of Trauma and Orthopaedics, Milton Keynes University Hospital NHS Foundation Trust, Eaglestone, Milton Keynes, Buckinghamshire, UK; e Department of Medicine for Older People and Department of Diabetes, Warrington and Halton Teaching Hospitals NHS Foundation Trust, Warrington, UK; f Faculty of Medicine and Health Sciences, University of Buckingham, UK; g These authors contributed equally to the case report.

**Keywords:** Chilaiditi syndrome, Pregabalin, Postoperative periods

## Abstract

Chilaiditi syndrome is characterized by the interposition of the colon between the liver and diaphragm, leading to various abdominal symptoms. Current literature explores the suspected etiology of this condition including diaphragmatic conditions such as phrenic injury or deficient peritoneal attachments that lead to laxity of suspensory ligaments or enlarged subdiaphragmatic space; however, there is considerable ambiguity in the contributing factors to the development of Chilaiditi syndrome due to its rarity. Furthermore, an association between Chilaiditi syndrome and psychiatric conditions has been found in the literature but the evidence base is limited due to paucity of cases. We present a case involving an 85-year-old female patient who was initially admitted with hip pain, later found to have a neck of femur fracture, and underwent hemiarthroplasty. The patient’s past medical history was significant for depression, generalized anxiety disorder, pulmonary embolism, osteopenia, osteoarthritis, and a previous stroke. Postoperatively, the patient exhibited abdominal distention and absent bowel movements. A chest X-ray showed gas-filled loops in both hemidiaphragms, and computed tomography (CT) imaging identified the anterior interposition of bowel segments into the liver. The patient had been prescribed pregabalin as part of the treatment for her psychiatric conditions. Although no link has been established between the development of Chilaiditi syndrome and the use of pregabalin in current literature, it is worth noting that pregabalin’s side effect profile includes gastrointestinal symptoms such as abdominal distention and constipation, similar to those seen in Chilaiditi syndrome. Further research is needed to assess the potential association between pregabalin and Chilaiditi syndrome. As well as considering pregabalin’s possible contribution to the development of Chilaiditi syndrome, it is worth considering psychological conditions as a risk factor for development of Chilaiditi syndrome, independent of medications. There is an established link which already exists, but the reasons as to why this exists are yet to be cemented. Similarly, few cases of Chilaiditi syndrome developing in the perioperative setting have been reported, so this case is valuable in considering the role of anesthetics and postoperative analgesia in the development of this syndrome.

## Introduction

In this case report, we present an 85-year-old female presenting with Chilaiditi syndrome. Chilaiditi sign is a radiological finding which refers to the hepatodiaphragmatic interposition of the colon [1]. Anterior interposition of the colon between the liver and the diaphragm is normally prevented by the presence of suspensory ligaments as well as fixation of the colon [2]. Hepatic flexure, transverse colon, and ascending colon are most interposed into the liver, but portions of small bowel can also get interposed [2]. Chilaiditi sign is a rare radiological finding with an estimated incidence of 0.025% to 0.28% [3]. Males have an increased prevalence over females particularly over the age of 60 years [3, 4]. It is critical to differentiate between Chilaiditi and pneumoperitoneum to avoid unnecessary surgical intervention. To diagnose Chilaiditi based on radiography, certain criteria need to be met in erect position: bowels must be distended by air, the right hemidiaphragm must be elevated from the liver by the intestine, and the liver must be depressed below the left hemidiaphragm [2].

Although etiology of this condition remains unclear, it is suspected that diaphragmatic conditions such as phrenic injury or deficient peritoneal attachments that lead to laxity of suspensory ligaments or enlarged subdiaphragmatic space might lead to development of Chilaiditi [3]. Other factors that can predispose one to develop Chilaiditi syndrome that influence the reasons aforementioned can include congenital malposition, functional disorders such as chronic constipation (caused by colonic elongation and redundancy), gaseous distension of the colon, small liver due to cirrhosis or resection, ascites due to increased intra-abdominal pressure, substantial weight loss, a chronic obstructive lung disease that causes enlargement of the lower thoracic cavity, and multiple pregnancies [5]. An association between psychiatric conditions, mental retardation, and Chilaiditi has been reported in the literature [6, 7]. This case report aims to supplement the limited available literature on Chilaiditi syndrome, explore its possible links with psychiatric conditions, pregabalin use, and perioperative setting factors such as use of anesthetic and postoperative analgesia, evaluate their role in the development of this condition, assess the strength of existing evidence, and address identified gaps in the current literature.

## Case Report

An 85-year-old female presented to the emergency department with a presenting complaint of sharp shooting left hip pain. The pain was localized to the left hip and began the night prior when the patient attempted to get out of the bath; the pain got progressively worse over the next day and the patient was unable to walk due to the pain. The patient reported no recent weight loss and no night sweats. The past medical history was significant for agitated depression with multiple admissions into the hospital, generalized anxiety, pulmonary embolism, stroke, chronic kidney disease stage 3, osteoarthritis, and osteopenia. She was on pregabalin and lithium carbonate for management of her psychiatric conditions.

**Figure 1:**
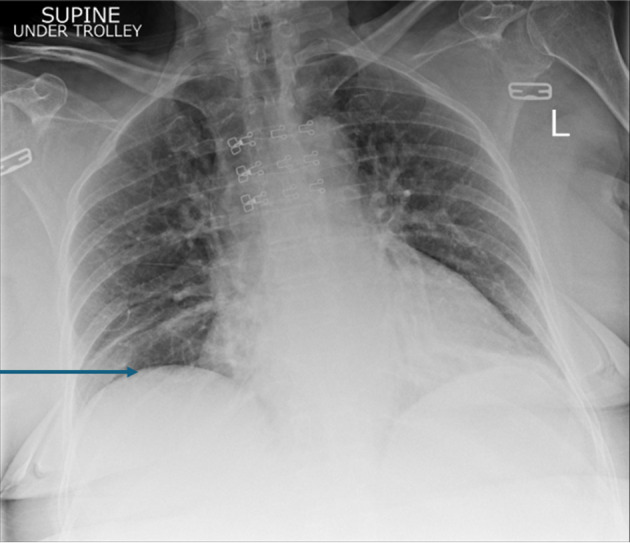
Initial supine chest radiograph (CXR) revealed shallow lung expansion with elevated hemidiaphragms more in the right side. The arrow points towards elevated right hemidiaphragm. Image taken preoperatively.

**Figure 2:**
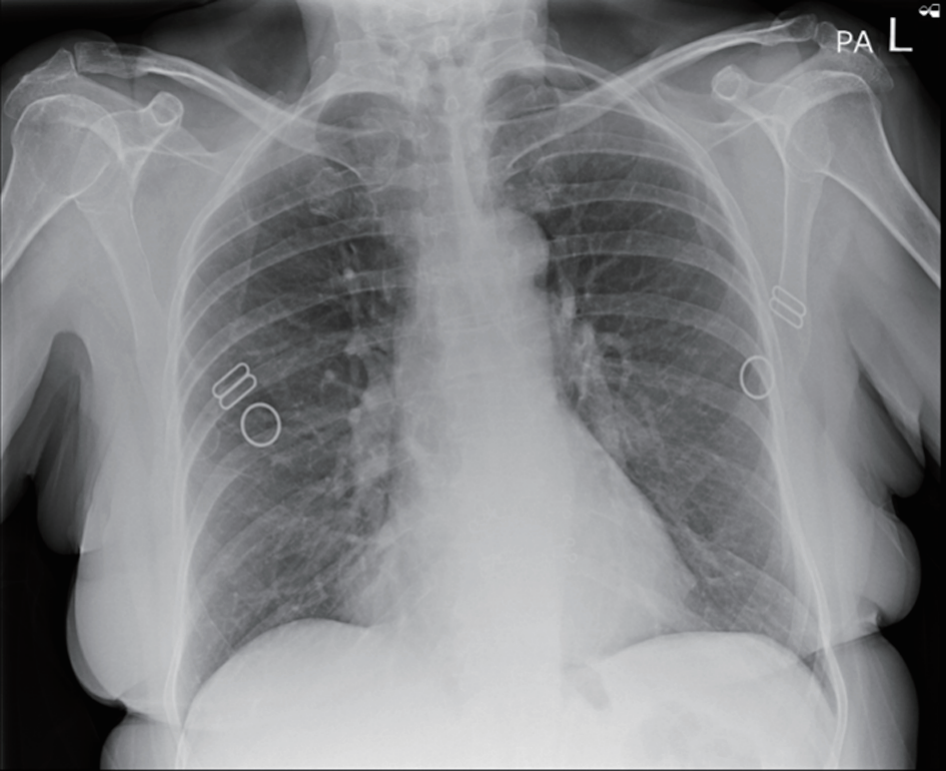
Anterior-posterior chest X-ray, 3 years prior to presentation showing normal chest X-ray and no gas loops in the hemidiaphragm.

**Figure 3:**
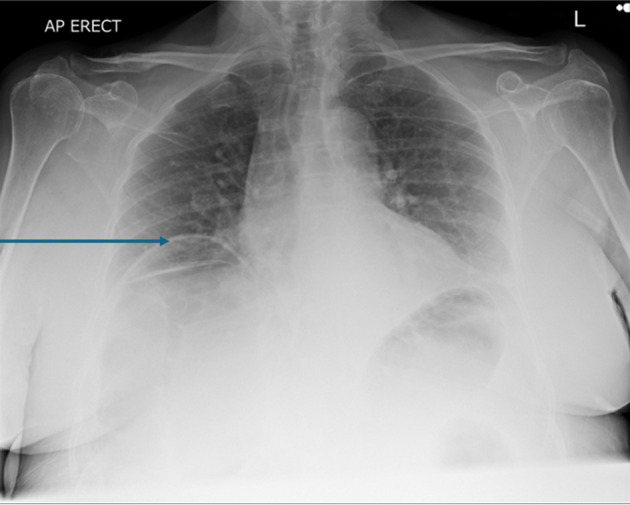
Anterior-posterior chest X-ray on the day of symptoms beginning showing bilateral gas filled loops beneath both hemidiaphragms more in the right side. The arrow points towards elevated right hemidiaphragm. Images taken day 1 postoperatively.

**Figure 4:**
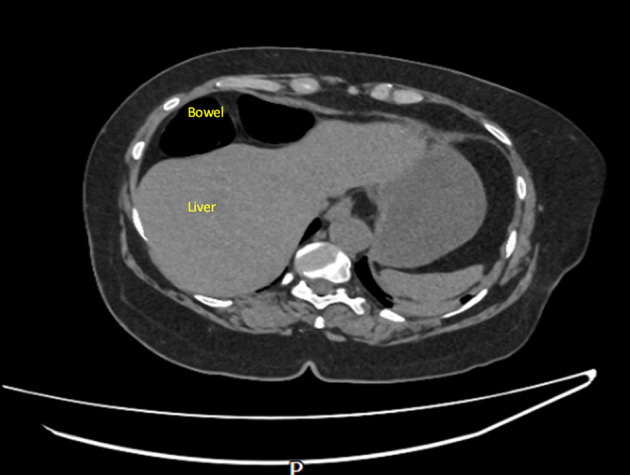
Confirmed Chilaiditi, normal variant with normal appearances of the solid abdominal organs. No dominant lymphadenopathy. No free fluid. No pelvic mass. Images taken day 1 postoperatively.

On examination, the patient was found to have externally rotated and shortened left leg and bilateral lower leg edema. Initial abdomen examination revealed soft non-tender abdomen. X-ray imaging of pelvis revealed subcapital fracture of the left neck of femur. She was afebrile (36.4 °C) with heart rate of 68 bpm, oxygen saturation of 97%, and blood pressure of 170/89 mm Hg. On auscultation, the chest was clear bilaterally with a midline trachea and no crepitus. The patient was subsequently diagnosed with non-traumatic neck of femur fracture and underwent hemiarthroplasty of the left leg under general anesthetic. Postoperative examination of the patient revealed soft non-tender but distended abdomen with absent bowel movement. The patient reported no discomfort, but it remains unclear whether this is due to postoperative analgesia or her continuing pregabalin use. Her postoperative analgesia included codeine 30 mg four times a day with breakthrough oral morphine 10 mg as required. The patient opened her bowels on postoperative day 2. Initial supine chest radiograph (CXR) revealed shallow lung expansion with elevated hemidiaphragms more in the right side (Fig. 1), compared with CXR performed 3 years ago which was normal (Fig. 2). Repeat anterior-posterior chest radiograph conducted the second day postoperatively showed gas filled loops of bowel beneath the hemidiaphragms with a distended bowel (Fig. 3). Although pneumoperitoneum was the primary concern, it was crucial to rule this out using both clinical and imaging studies. It remained unclear whether a small bowel obstruction or an abscess was present in X-ray. Computed tomography (CT) was conducted which revealed interposition of bowel between the liver and diaphragm which is consistent with Chilaiditi sign and small compressive atelectasis in the lung basis. The CT scan ruled out the presence of any abnormal bowel dilation, pneumoperitoneum, and abdominal mass (Fig. 4). The patient received medical optimization for bone protection, intensive physiotherapy, and occupational therapy input. She was discharged 13 days postoperatively with twice-daily care support. Reports in her 3-month outpatient orthopedic review remarked on her good recovery. No specific follow-up for her Chilaiditi syndrome was required and her recovery from this was uneventful.

## Discussion

Chilaiditi sign is a radiological sign that is incidentally found in patients that present asymptomatically. However, when patients present with abdominal distention, constipation, vomiting, and abdominal pain similar to our patient, it is known as Chilaiditi’s syndrome [2]. Our patient lacked abdominal pain but this was most likely in relation to her postoperative analgesia (regular codeine and morphine as required) as well as her ongoing pregabalin use which likely masked this abdominal pain [8]. In our case, the confounding factor of being postoperative along with the host of postoperative medications (most notably analgesia), made a diagnosis of Chilaiditi syndrome more difficult as some degree of constipation may be expected when reliant on opioid medication in this particular setting, so a CT was crucial in differentiating fecal impaction as a side effect versus Chilaiditi syndrome. Furthermore, the role of undergoing an anesthetic alongside the abundant postoperative analgesia required after a hemiarthroplasty in the development of Chilaiditi syndrome is unknown and requires considerable further investigation to clarify their contribution. CT alongside clinical examination, including digital rectal examination, provides the best means to aid a diagnosis. A prevalence of 0.025% to 0.28% of Chilaiditi has been reported in the general population with an increased prevalence in men [3]. A retrospective study analyzing abdominal CT scans of 1,440 patients with 806 male and 634 female reported the actual incidence to be 2.4% and 1.8% respectively in general population [9]. In the general population, an increased prevalence is also observed in the geriatric population with a retrospective study observing a 1%, with a fourfold increase in males. An incidence rate of 0.147% to 1.000% has been reported in patients diagnosed with mental retardation and psychiatric conditions associated with psychosis [6].

Matsuo et al linked the increased prevalence of Chilaiditi syndrome in patients with mental disorders to increased tendency of this patient group to suffer from constipation and meteorism [10]. This theory would support Choussat and Choussat-Clausse’s study that analyzed and categorized the causes of this interposition to be related to either hepatic factor (small or ptosis liver), diaphragmatic factors (intrathoracic pressure change, phrenic nerve injury) or intestinal factors (accumulation of gas in the colon resulting in upward mobility of the colon and displacement of the diaphragm) [11]. The increased prevalence of Chilaiditi syndrome has also been linked to the use of antipsychotics in this patient group [12]. Antipsychotics drugs have been associated with side effects such as ileus and constipation [13]. The patient in this case study has a significant past medical history of mental health disorders, including depression and generalized anxiety disorder, which may be contributing factors to the development of this interposition. As part of the management for this condition, the patient was also prescribed pregabalin. Although no link has been established between developing Chilaiditi syndrome and the use of pregabalin in current literature, it is worth noting that pregabalin’s side effect profile contains gastrointestinal symptoms such as abdominal distention and constipation similarly to Chilaiditi syndrome [13]. It is also worth noting that the interposition was noted only in the postoperative imaging studies, which prompts us to question whether factors such as anesthetic use, use of muscle relaxants in surgery, and reduced mobility in this patient group lead to the development of Chilaiditi syndrome.

Differential diagnosis includes subphrenic abscess, pneumoperitoneum or a right-sided diaphragmatic hernia. CT imaging can be used to distinguish between true pneumoperitoneum, abscess, and Chilaiditi syndrome by altering the patient’s position; the “free gas” loops will remain in the hepatodiaphragmatic recess in Chilaiditi [14]. Additionally, identifying haustral marking and plicae circulares in CT scans should also help to exclude these differentials [14]. Differentiating between these conditions is crucial as the treatment normally involves invasive explorative laparotomy. Chilaiditi syndrome can be conservatively managed by intravenous fluid, high fiber diet, and stool softeners as a first line. Complication or persistence of symptoms is an indication for surgery [6]. This case report acts as a further addition to the limited case report repertoire that currently exists surrounding the rare Chilaiditi syndrome. It briefly highlights various possible contributing factors (learning points) to the development of this syndrome which include pre-existing psychological diagnoses, pregabalin use, and the administration of anesthetic and extensive postoperative analgesia in the perioperative setting. It is difficult to determine which factors played the most significant role given their interrelated nature. However, further research into the aforementioned factors is clearly warranted, as misdiagnosis of this syndrome can lead to excessive investigations and unnecessary surgeries. These not only impair patients’ quality of life but also impose avoidable economic burdens on already strained healthcare systems.

### Conclusion

We presented a rare case of Chilaiditi syndrome mimicking postoperative pneumoperitoneum. This case highlights the complexities surrounding a discovery of Chilaiditi syndrome in a postoperative patient with a background of psychiatric illness. Further research is needed to assess the potential association between pregabalin and Chilaiditi syndrome. Further research is also warranted to explore the development of Chilaiditi syndrome in the perioperative setting and to determine the role of recent anesthetic administration and postoperative analgesia in its pathogenesis. This research is critical in preventing unnecessary investigations and surgical interventions and can be avoided by obtaining a CT of abdomen pelvis to clarify concerning CXR findings.

## Data Availability

The authors declare that data supporting the findings of this study are available within the article.
